# Le syndrome de sevrage nicotinique après chirurgie cardiaque: à propos d’un cas

**Published:** 2012-09-13

**Authors:** Samy Kallel, Maged Ellouze, Zied Triki, Abdelhamid Karoui

**Affiliations:** 1 Service d’Anesthésie Réanimation Chirurgicale, CHU Habib Bourguiba, Sfax, Tunisie

**Keywords:** Agitation post opératoire, chirurgie cardiaque, syndrome de sevrage nicotinique, postoperative agitation, cardiac surgery, nicotine withdrawal syndrome

## Abstract

L’agitation post-opératoire constitue une pathologie extrêmement fréquente. Les étiologies à évoquer en réanimation sont nombreuses. Le syndrome de sevrage à la nicotine est une cause possible mais rarement évoquée. Nous rapportons le cas d’un patient tabagique qui a été admis en unité de soins intensifs pour un triple pontage aorto-coronaire sous circulation extracorporelle. Les suites opératoires ont été marquées par des difficultés de sédation du patient et puis la survenue d’une agitation au réveil et d’un échec du sevrage de la ventilation mécanique. Après avoir éliminer toutes les causes organiques d’agitation, le syndrome de sevrage à la nicotine a été évoqué et la mise en place d’un timbre de nicotine a permis une amélioration rapide de l’agitation et le sevrage de la ventilation mécanique. Cette observation suggère que le syndrome de sevrage nicotinique peut être responsable d’un véritable delirium et la mise en place d’un timbre transdermique à la nicotine pourrait constituer un moyen diagnostique et thérapeutique simple.

## Introduction

L’agitation post-opératoire constitue une pathologie extrêmement fréquente que l’on peut définir comme une hyperactivité motrice quasi permanente, spontanée ou réactionnelle, sans mobile apparent [1]. L’agitation post-opératoire peut survenir en salle de surveillance post-interventionnelle au décours immédiat d’une intervention chirurgicale ou après un intervalle libre de plusieurs jours en unité de soins chirurgicaux ou en réanimation. Les étiologies à évoquer devant une agitation, une sédation difficile ou un échec de sevrage de la ventilation mécanique en réanimation sont presque les mêmes, mais leur ordre hiérarchique est dépendant du contexte. Le syndrome de sevrage à la nicotine est une cause possible mais rarement évoquée.

## Patient et observation

Nous rapportons le cas d’un patient âgé de 45 ans sans antécédents pathologiques particuliers, admis en unité de soins intensifs pour réanimation post-opératoire d’un triple pontage aorto-coronaire. Les explorations qui ont été réalisées en préopératoire ont montré une bonne fonction ventriculaire gauche systolique et diastolique à l’échographie cardiaque. La coronarographie a objectivé une sténose significative du tronc commun gauche, une double sténoses serrées de l’IVA distale, une sténose très serrée de la CDII et une sténose serrée de la CD III et le doppler des troncs supra aortiques était sans anomalie.

Dans ses habitudes, nous avons noté l’absence stricte de consommation d’alcool, une consommation de 40 cigarettes par jour avec une intoxication tabagique évaluée à 50 paquets.année. Les durées de la chirurgie, de la CEC et du clampage aortique ont été respectivement de 240 min, 100 min et 60 min.

Le patient a été extubé à H2 post-opératoire (PO) et sevré des catécholamines à j1PO. L’évolution a été marquée à j2 PO par l’installation d’un état de choc septique a point de départ pulmonaire nécessitant le recours à la ventilation mécanique avec une sédation. Une TDM thoracique a été en faveur d’une pneumopathie et a montré un emphysème pan-lobulaire diffus aux 2 poumons plus marqué à droite.

Le traitement initial comprenait une stabilisation hémodynamique par des vasoconstricteurs, une antibiothérapie probabiliste et une nutrition entérale précoce. Malgré des doses de 0,15 mg/kg par heure de midazolam et de 4 µg/kg par heure de fentanyl, le niveau de la sédation était insuffisant avec un score de Ramsay inférieur à 3.Une désadaptation au respirateur et des épisodes d’agitation ont marqué la période de sédation du patient. Devant la perspective d’une ventilation prolongée et d’un sevrage ventilatoire difficile, une trachéotomie chirurgicale a été réalisée à j 10 PO.

Le sevrage de la ventilation mécanique a été débuté à j12 après l’obtention de prés requis ventilatoires (FiO2= 40%; PEP = 3; PaO2/FiO2 = 260) et généraux [2]. A j 15, l’amélioration du niveau de conscience a été accompagnée de l’apparition d’hallucinations visuelles et de difficultés de concentration et d’une agitation importante responsable de l’échec du sevrage ventilatoire. Après avoir éliminé une hypoxie, un désordre métabolique ou hydro-électrolytique et un globe vésical, un infarctus cérébral en post-CEC par un scanner cérébral normal et un delirium tremens chez ce patient qui n’a jamais consommé d’alcool, le syndrome de sevrage aux benzodiazépines et aux morphiniques a été évoqué dans un premier temps et de ce fait nous avons administré par voie intra veineuse du tranxéne 20mg, de l’halopéridol 30 mg et de la clonidine 450 mg. Après 48 heures devant la non amélioration, le syndrome de sevrage à la nicotine a été évoqué et un timbre transdermique de 21mg de nicotine a été mis en place. En quelques heures, nous avons observé un arrêt de l’agitation et des hallucinations ainsi que la reprise d’une ventilation calme et adaptée au respirateur. Le sevrage de la ventilation mécanique a été réalisé avec succès et le patient a été décanulé à j + 24. A J+ 27 le patient a été transféré au service d’hospitalisation.

## Discussion

L’arrêt de la sédation s’accompagne souvent de manifestations cliniques dont il est très difficile de distinguer si elles sont la conséquence d’une intolérance médicamenteuse, d’un syndrome de sevrage ou de l’existence de troubles centraux non diagnostiqués pendant la phase de sédation.

Le syndrome de sevrage en réanimation reste un diagnostic d′élimination une fois que toutes les causes organiques d′agitation/confusion ont été exclues. La chirurgie cardiaque avec circulation extracorporelle est connue comme un facteur favorisant la survenue de délire et d’agitation post-opératoire. Le délire peut compliquer un accident vasculaire cérébral embolique dans près de la moitié des cas [3].

Le syndrome de sevrage alcoolique est une pathologie fréquente et un facteur de morbi-mortalité en l′absence de traitement efficace. Le syndrome de sevrage aux morphiniques ou aux benzodiazépines en réanimation est pourvoyeur de morbidité par le biais d′une prolongation de la durée de la ventilation mécanique. La consommation régulière de tabac s’accompagne d’une dépendance psychique et surtout physique. La substance considérée comme la principale impliquée dans la survenue de cette dépendance est la nicotine. L’existence d’un syndrome de sevrage nicotinique témoigne de cette dépendance pharmacologique et se traduit dans les heures qui suivent l’arrêt brutal ou la diminution significative de la consommation [4].

Chez les patients hospitalisés, l’intensité est variable selon les fumeurs. Il peut s’exprimer par la survenue de symptômes minimes tels l’existence de besoins impérieux de fumer, de nervosité, d’anxiété, d’irritabilité, d’impatience, de baisse des performances et de difficultés de concentration. Toutefois, des formes graves associant une agitation, une agressivité, une confusion et des hallucinations peuvent se voir.

L’observation que nous rapportons illustre les problèmes spécifiques posés par le sevrage nicotinique en réanimation. Pour expliquer l’état d’agitation qui a été présenté par ce patient, des causes simples et immédiatement curables ont été éliminées. Le possible survenu d’accident vasculaire cérébral par le biais de microembols suite à la CEC a été infirmée par le scanner cérébral. Un syndrome de sevrage aux benzodiazépines ou aux morphiniques a été écarté devant l’absence d’amélioration malgré la diminution progressive des doses de midazolam et de fentanyl et l’administration d’halopéridol, de tranxéne et de clonidine.

L’arrêt très rapide du delirium après la mise en place d’un timbre de nicotine a confirmé l’hypothèse d’un syndrome de sevrage nicotinique que nous avions évoqué chez ce patient gros fumeur. La fréquence de survenue d’un syndrome de sevrage est difficile à préciser mais probablement sous-estimée. Ce syndrome a déjà été décrit dans la littérature. Le sevrage aigu à la nicotine a été incriminé dans des tableaux de délire aigu en neuroréanimation [5]. Dans plusieurs cas graves publiés, la disparition des signes était rapide, quelques heures après la mise en place d’un timbre de nicotine [6]. En réanimation, une série de cinq cas a été publiée par une équipe de soins intensifs neurologiques américaine [5]. Il s’agit de patients souffrant d’accident vasculaire cérébral et d’hémorragie méningée, qui ont présenté une agitation et une confusion deux à dix jours après l’arrêt du tabac. Dans les cinq cas, la mise en place d’un timbre de nicotine délivrant 21 mg par 24 heures a permis une amélioration rapide de la situation. Pour les patients fumeurs admis en réanimation, le syndrome de sevrage à la nicotine existe vraisemblablement car l’arrêt du tabac est complet et brutal. Une étude prospective incluant des patients de réanimation a montré une agitation importante associée à une augmentation de la morbidité chez les patients tabagiques ayant subi un arrêt brutal de tabac comparés aux patients non tabagiques, le syndrome de sevrage nicotinique a été mis en cause. [7]. Chez les patients ventilés, les difficultés de sédation et l’agitation au réveil posent souvent un problème diagnostique. Pour les patients alcoolo tabagiques, c’est le delirium tremens qui est le plus fréquemment incriminé à défaut du syndrome de sevrage nicotinique qui reste méconnu. Cependant, le diagnostic de ce syndrome pourrait être facilité par la pose d’un timbre transdermique de nicotine qui constitue un test diagnostic et un moyen thérapeutique. Facile d’utilisation et peu onéreux, il permet d’atteindre un taux plasmatique de nicotine suffisant pour réduire le syndrome de sevrage en quatre à cinq heures [4].

En réanimation, les réticences à son utilisation résident vraisemblablement dans les complications cardiovasculaires sévères rapportées par quelques auteurs [8–10]. Toutefois, une méta-analyse [11] et deux études prospectives [12, 13] publiées depuis ont démontré que le timbre de nicotine n’augmente le risque cardiovasculaire en particulier chez le coronarien stable. Cependant une revue approfondie de la littérature ne trouve pas d’étude randomisée contrôlée large qui étudie la sécurité de la substitution nicotinique chez le coronarien instable, pendant la phase aiguë ou en post revascularisation coronaire. Ainsi serait- il difficile de recommander la pose systématique d’un patch de nicotine chez les grands fumeurs après chirurgie coronarienne. Cependant la substitution serait nécessaire devant une agitation avec des difficultés le sevrage ventilatoire et mettant en jeu le pronostic vital du patient.

## Conclusion

Il convient d’être vigilant lors de la période postopératoire pour diagnostiquer un syndrome de sevrage tabagique qui peut apparaître chez des patients gros fumeurs et sevrés brutalement sans substitution nicotinique. Ce syndrome peut être responsable d’un véritable delirium et aggraver le pronostic de ces malades. La mise en place d’un timbre transdermique à la nicotine pourrait constituer un moyen diagnostique et thérapeutique simple et efficace permettant la régression rapide de la symptomatologie.
